# INCIDENCE OF PEDESTRIAN TRAFFIC INJURY IN SÃO PAULO, BRAZIL, IN 2016

**DOI:** 10.1590/1413-785220182602185837

**Published:** 2018

**Authors:** RODRIGO TADASHI MARTINES, WASHINGTON GOMES DE ARAUJO, CINTIA LECI RODRIGUES, JANE DE ESTON ARMOND

**Affiliations:** 1. Department of Collective Health, School of Medicine, Universidade de Santo Amaro, Sao Paulo, SP, Brazil.

**Keywords:** Pedestrians, Accidents, traffic, Wounds and injuries, Multiple trauma, Automobile driving, Cross-sectional studies., Pedestres, Acidentes de trânsito, Ferimentos e lesões, Traumatismo múltiplo, Condução de veículo, Estudos transversais.

## Abstract

**Objective:**

Globally, more than 1.2 million people die from traffic accidents each year. In order to reduce the rate of traffic accidents and their subsequent social consequences in Sao Paulo, Brazil, the aim of this study was to characterize pedestrian victims of traffic accidents and occurrences.

**Methods:**

This is a cross-sectional, quantitative, descriptive and retrospective epidemiological study of 2723 pedestrians injured in traffic accidents during 2016 in Sao Paulo.

**Results:**

Of the total sample, 37.3% participants were female and 62.7% male; incidence was highest in the 10-19 year old age group (19.9%) and lower limb injuries were most frequent (28.6%). Of the cases attended in urgent health care units, 75.6% progressed to hospital discharge. Accidents were more frequent in the afternoon (44.6%), and most commonly involved cars (47.2%).

**Conclusions:**

This study showed the importance of making detailed records of traffic accidents to guide the government in intensive education efforts to develop a healthy society and respect for traffic regulations, to promote urban improvements involving signage and pavement, and to maintain pre-hospital and hospital care teams in strategic locations to minimize the time elapsed between the accident and care provided to the victim. Level of Evidence II; Retrospective study.

## RESUMO

**Objetivo:**

Mundialmente, mais de 1,2 milhão de pessoas morrem por ano vítimas de acidentes de trânsito. Visando a redução das taxas de acidentes de trânsito e suas consequências sociais em São Paulo, Brasil, o objetivo deste estudo foi caracterizar as vítimas de acidentes e ocorrências de trânsito.

**Métodos:**

Este é um estudo epidemiológico transversal, quantitativo, descritivo e retrospectivo de 2.723 pedestres vítimas de acidentes de trânsito durante 2016 em São Paulo.

**Resultados:**

Do total da amostra, 37,3% participantes eram mulheres e 62,7% homens; a incidência foi maior no grupo de 10 a 19 anos (19,9%) sendo as lesões de membros inferiores as mais frequentes (28,6%). Dos casos encaminhados às unidades de pronto atendimento, 75,6% evoluíram para alta hospitalar. Os acidentes foram mais frequentes no período da tarde (44,6%) e a maioria associada a automóveis (47,2%).

**Conclusões:**

O estudo mostrou a importância de realizar o registro detalhado de acidentes de trânsito para orientar os governantes para a educação intensiva da população visando o desenvolvimento de uma sociedade sadia e o respeito às leis de trânsito, promover melhorias urbanas quanto à sinalização e ao pavimento das vias e para manter equipes de atendimento pré-hospitalar e hospitalar em locais estratégicos para minimizar o intervalo de tempo entre o acidente e o atendimento das vítimas. Nível de Evidência II; Estudo retrospectivo.

## INTRODUCTION

The world economic growth has facilitated the increase of vehicle circulation, which makes traffic more complex and dangerous.^1^ According to the Brazilian Traffic Code (1997), traffic is defined as the use of lanes by people, vehicles and animals isolated or in groups, driven or not, for the purposes of circulating, parking and loading or unloading vehicles.^2^

Globally, more than 1.2 million people die from traffic accidents each year and it represents the fifth leading cause of death.^1^ In Brazil, external causes are ranked in the third position. In this country, 8200 traffic accidents with pedestrians were recorded in 2013, which is equivalent to a rate of 4.1 per 100 000 inhabitants. Among the reasons for traffic injuries and deaths are structural factors such as roads and urban lanes maintenance, increased fleet of vehicles, use of alcohol associated with driving and non-use of safety equipment.^3^ Due to the vulnerability of pedestrians, the traffic accidents represent an important cause of morbidity and mortality in Brazil.^4^

Traffic accidents produce high emotional and social costs for victims and their families. They are able to generate mental damage, days of absence and decrease of productivity from work.^5^ Psychological consequences and posttraumatic stress disorders resulting from ground transportation accidents are potentially disabling in the long-term.^6^

In epidemiology, traffic accidents have different distributions for gender, age, social groups and areas of risk, indicating situations of vulnerability of people and places.^7^ ^-^ ^9^ The characterization of the traffic accidents and the victims contribute to urban planning and to the implementation of effective strategies in the prevention of accidents by the government.^10^

In 2010, the United Nations (UN) proclaimed the period from 2011 to 2020 as the Decade of Action for Traffic Safety. In this period countries should stabilize and reduce traffic fatalities by implementation of an action plan focused on the five pillars of interventions: road safety management, safer roads and mobility, safer vehicles, safer road users, and post-crash response. Therefore, the Brazilian Ministry of Health implemented the Transit Life Project in 2010 with focus on the strengthening of policies for injury prevention and death in traffic, through qualification of information, planning, monitoring and interventions evaluation.^11^

Faced with the challenges of reducing the number of traffic accidents and their social consequences, and for adequacy of pre-hospital and hospital care of trauma in the city of Sao Paulo, the aim of this study was to characterize the pedestrians injured in traffic accident in regard to gender, age, period of occurrence, association with alcohol and drug use, location of accidents, trauma diagnosis, medical evolution of the victims and vehicles involved, in order to guide the government in urban and hospital development strategies.

## METHODS

This study is a cross-sectional, quantitative, descriptive and retrospective epidemiological study of pedestrians injured in traffic accident records, during 2016 in the city of Sao Paulo, state of Sao Paulo, Brazil.

Secondary data was collected on July 31, 2017 through the Violence and Accident Surveillance Information System (SIVVA) in the city of Sao Paulo, managed by the Health Surveillance Coordination of the Municipal Health Secretary of Sao Paulo (COVISA - SMS) in conjunction with Health Surveillance Supervisors (SUVIS). It analyzed notifications of traffic accidents involving pedestrians during the period from January 1, 2016 to December 31, 2016.

The group study consisted of 2723 pedestrian victims of traffic accidents. The variables were distributed as follows: characteristics of victims regarding gender (male and female), age group (in years), alcohol and drug use, main trauma diagnosis and medical evolution of the victims, characteristics of occurrences regarding time of the day (morning, afternoon, night and early hours), urban area (north, south, east, west, southeast and central) and types of vehicles (car, motorcycle, bicycle, heavy vehicle and others) involved in accidents.

After data collection, they were tabulated and processed in EXCEL software. Afterwards, descriptive statistical analyzes were performed, calculating the absolute and relative frequencies of variables.

According to the recommendation of the Resolution 466/12 of the Brazilian National Health Council (CNS) this work didn’t need approval by the University Ethics Committee, since secondary data was used and was available in a public domain database.

## RESULTS

According to the records from the SIVVA, in Sao Paulo during 2016, there were 2723 pedestrians injured in traffic accidents, 1017 (37.3%) female and 1706 (62.7%) male. It was observed that there was a higher incidence of accidents in 1the age group of 15-19 years (12.3%), followed by 20-24 years (11.0%) and 5-9 years (9.4%). Regarding alcohol consumption, 59 (2.2%) victims were under the effect of alcohol. As for illicit drugs use, only 17 (0.6%) pedestrians were under the influence of narcotics. (Table 1)

**Table 1:** Distribution of pedestrian victims of traffic accident. according to age, gender, alcohol and drug use in São Paulo, 2016.

Age (years)	Female N (%)	Male N (%)	Alcohol (+) N (%)	Alcohol (-) N (%)	Alcohol ignored N (%)	Drugs (+) N (%)	Drugs (-) N (%)	Drugs ignored N (%)	Total N (%)
0 a 4	71 (2.6)	136 (5.0)	0 (0)	60 (2.2)	147 (5.4)	0 (0.0)	58 (2.1)	149 (5.5)	207 (7.6)
5 a 9	77 (2.8)	178 (6.5)	0 (0)	88 (3.2)	167 (6.1)	0 (0.0)	88 (3.2)	167 (6.1)	255 (9.4)
10 a 14	71 (2.6)	135 (5.0)	1 (0.0)	64 (2.4)	141 (5.2)	1 (0.0)	64 (2.4)	141 (5.2)	206 (7.6)
15 a 19	137 (5.0)	199 (7.3)	4 (0.1)	100 (3.7)	232 (8.5)	2 (0.1)	102 (3.7)	232 (8.5)	336 (12.3)
20 a 24	98 (3.6)	202 (7.4)	10 (0.4)	103 (3.8)	187 (6.9)	7 (0.3)	103 (3.8)	190 (7.0)	300 (11.0)
25 a 29	60 (2.2)	126 (4.6)	5 (0.2)	59 (2.2)	122 (4.5)	1 (0.0)	60 (2.2)	125 (4.6)	186 (6.8)
30 a 34	57 (2.1)	113 (4.1)	6 (0.2)	65 (2.4)	99 (3.6)	3 (0.0)	65 (2.4)	102 (3.7)	170 (6.2)
35 a 39	56 (2.1)	113 (4.1)	7 (0.3)	63 (2.3)	99 (3.6)	0 (0.0)	69 (2.5)	100 (3.7)	169 (6.2)
40 a 44	46 (1.7)	93 (3.4)	7 (0.3)	49 (1.8)	83 (3.0)	2 (0.1)	49 (1.8)	88 (3.2)	139 (5.1)
45 a 49	54 (2.0)	86 (3.2)	3 (0.1)	43 (1.6)	94 (3.5)	0 (0.0)	44 (1.6)	96 (3.5)	140 (5.1)
50 a 54	60 (2.2)	79 (2.9)	5 (0.2)	39 (1.4)	95 (3.5)	0 (0.0)	42 (1.5)	97 (3.6)	139 (5.1)
55 a 59	57 (2.1)	68 (2.5)	6 (0.2)	54 (2.0)	65 (2.4)	1 (0.0)	57 (2.1)	67 (2.5)	125 (4.6)
60 a 64	54 (2.0)	66 (2.4)	1 (0.0)	47 (1.7)	72 (2.6)	0 (0.0)	48 (1.8)	72 (2.6)	120 (4.4)
65 a 69	35 (1.3)	30 (1.1)	2 (0.1)	31 (1.1)	32 (1.2)	0 (0.0)	34 (1.2)	31 (1.1)	65 (2.4)
70 a 74	38 (1.4)	28 (1.0)	2 (0.1)	23 (0.8)	41 (1.5)	0 (0.0)	23 (0.8)	43 (1.6)	66 (2.4)
75 a 79	22 (0.8)	18 (0.7)	0 (0)	18 (0.7)	22 (0.8)	0 (0.0)	18 (0.7)	22 (0.8)	40 (1.5)
80 a 84	15 (0.6)	24 (0.9)	0 (0)	11 (0.4)	28 (1.0)	0 (0.0)	11 (0.4)	28 (1.0)	39 (1.4)
85 a 89	8 (0.3)	6 (0.2)	0 (0)	6 (0.2)	8 (0.3)	0 (0.0)	6 (0.2)	8 (0.3)	14 (0.5)
90 e +	1 (0.0)	5 (0.2)	0 (0)	1 (0.0)	5 (0.2)	0 (0.0)	1 (0.0)	5 (0.2)	6 (0.2)
Blank/ Ignored	0 (0.0)	1 (0.0)	0 (0)	0 (0)	1 (0.0)	0 (0.0)	0 (0.0)	1 (0.0)	1 (0.0)
Total	1017 (37.3)	1706 (62.7)	59 (2.2)	924 (33.9)	1740 (63.9)	17 (0.6)	942 (34.6)	1764 (64.8)	2723 (100.0)

Source: Violence and Accident Surveillance Information System (SIVVA) - Health Surveillance Coordination of the Municipal Health Secretary of Sao Paulo (COVISA - SMS) - Health Surveillance Supervisors (SUVIS).

Table 2 shows the distribution of pedestrian victims of traffic accidents records by time of the day and urban area of the accidents. The time of the accidents was recorded in 985 (36.2%) cases with a higher incidence in the afternoon with 439 (44.6%), followed by 201 (20.4%) in the evening, 183 (18.6%) in the morning and 162 (16.4%) in early mornings. In urban area data, 393 (14.4%) occurrences registered the address of the accidents. Based on those records without the ignored location of the accidents, 116 (29.5%) occurred in the South, 103 (26.2%) in the Southeast, 102 (25.9%) in the East, 46 (11.7%) in the West, 21 (5.3%) in the North and 5 (1.2%) in the Central area. The other 2327 (85.6%) accidents had the address of occurrences ignored.

**Table 2:** Distribution of traffic accidents with pedestrians by periods and urban areas of occurrences in Sao Paulo, 2016.

Period / Urban area	Central N (%)	East N (%)	North N (%)	Southeast N (%)	South N (%)	West N (%)	Ignored N (%)	Total N (%)
Morning 7am - 12:59pm	3 (0.1)	16 (0.6)	4 (0.1)	13 (0.5)	19 (0.7)	12 (0.4)	116 (4.3)	183 (6.7)
Afternoon 1pm - 6:59pm	1 (0.0)	38 (1.4)	6 (0.2)	32 (1.2)	39 (1.4)	20 (0.7)	303 (11.1)	439 (16.1)
Evening 7pm - 12:59am	0 (0.0)	12 (0.4)	6 (0.2)	18 (0.7)	17 (0.6)	6 (0.2)	142 (5.2)	201 (7.4)
Early hours 1am - 6:59am	0 (0.0)	9 (0.3)	3 (0.1)	15 (0.6)	12 (0.4)	4 (0.1)	119 (4.4)	162 (5.9)
Blank/ Ignored	1 (0.0)	27 (1.0)	2 (0.1)	25 (0.9)	29 (1.1)	4 (0.1)	1650 (60.6)	1738 (63.8)
Total	5 (0.2)	102 (3.7)	21 (0.8)	103 (3.8)	116 (4.3)	46 (1.7)	2330 (85.6)	2723 (100.0)

Source: Violence and Accident Surveillance Information System (SIVVA) - Health Surveillance Coordination of the Municipal Health Secretary of Sao Paulo (COVISA - SMS) - Health Surveillance Supervisors (SUVIS).

The type of vehicle involved in the pedestrian traffic accidents is shown in Table 3. There were 1286 (47.2%) occurrences with cars, 630 (23.1%) with motorcycles, 157 (5.8%) with bicycles, 151 (5.5%) with heavy vehicles (buses, trucks and others). In 463 accidents (17.0%) the type of vehicle involved was not registered, considered blank or ignored.

**Table 3:** Distribution of types of vehicles involved in traffic accidents with pedestrians in Sao Paulo, 2016.

Type of vehicle	Notifications N (%)
Car	1286 (47.2)
Motorcycle	630 (23.1)
Bicycle	157 (5.8)
Bus/Truck/Other	151 (5.5)
Subway or Train	7 (0.3)
Air Transport	1 (0.0)
Other	28 (1.0)
Blank or Ignored	463 (17.0)
Total	2723 (100.0)

Source: Violence and Accident Surveillance Information System (SIVVA) - Health Surveillance Coordination of the Municipal Health Secretary of Sao Paulo (COVISA - SMS) - Health Surveillance Supervisors (SUVIS).

Based on the International Classification of Diseases 10 (ICD-10) and considering the different anatomical regions of the human body, lesions diagnosed in lower limbs (28.6%) were the most frequent. Head and neck injuries (20.7%), upper limbs (10.9%), chest (2.4%) and abdomen, back and pelvis (2.4%) followed, respectively, traumas in lower limbs. Regarding lesion types, contusions were predominant in 262 (9.6%) cases. The diagnosis was not recorded in 70 (2.57%) cases. The main diagnosis of injuries resulting from pedestrian traffic accidents can be observed in Table 4.

**Table 4:** Main diagnosis of trauma in pedestrian victims of traffic accidents in São Paulo, 2016.

ICD 10 and diagnosis	N (%)	% Anatomical region
Head and Neck		
S09.9 Unspecified injury of face and head	148 (5.4)	20.7
S00.9 Superficial injury of unspecified part of head	88 (3.2)	
S01.8 Open wound of other parts of head	58 (2.1)	
S09.8 Other specified injuries of head	57 (2.1)	
S01.9 Open wound of unspecified part of head	53 (1.9)	
Other ICDs of head and neck	161 (5.9)	
**Thorax**		
S20.2 Contusion of thorax	31 (1.1)	2.4
Other ICDs of thorax region	35 (1.3)	
**Abdomen, back and pelvis**		
S30.0 Contusion of lower back and pelvis	18 (0.7)	2.4
Other ICDs of abdomen, lower back and pelvis	47 (1.7)	
**Upper limbs**		
S40.0 Contusion of shoulder and upper arm	50 (1.8)	10.9
Other ICDs of upper limbs	248 (9.1)	
**Lower limbs**		
S80.0 Contusion of knee	104 (3.8)	28.6
S90.0 Contusion of ankle	59 (2.2)	
Other ICDs of lower limbs	615 (22.6)	
**Other diagnosis**		
T00.9 Multiple superficial injuries, unspecified	174 (6.4)	32.4
T07 Unspecified multiple injuries	168 (6.2)	
T14.9 Unspecified injuries	154 (5.7)	
Other ICDs of unspecified region	385 (14.1)	
Blank	70 (2.6)	2.6
Total	2723	100.0

Source: Violence and Accident Surveillance Information System (SIVVA) - Health Surveillance Coordination of the Municipal Health Secretary of Sao Paulo (COVISA - SMS) - Health Surveillance Supervisors (SUVIS)

Regarding care provided to pedestrian victims of traffic accidents, 2058 (75.6%) evolved to hospital discharge, 201 (7.4%) transferred to other health services, 95 (3.5%) needed hospitalization, and 20 (0.7%) resulted in death or died while in care. (Table 5)

**Table 5:** Medical evolution of the victims of traffic accidents in São Paulo in 2016.

Medical evolution	N (%)
Follow up	22 (0.8)
Discharge	2058 (75.6)
Observation	90 (3.3)
Hospitalization	95 (3.5)
Received in death	4 (0.1)
Transferred	201 (7.4)
Death in care	16 (0.6)
Blank or ignored	237 (8.7)
Total	2723 (100.0)

Source: Violence and Accident Surveillance Information System (SIVVA) - Health Surveillance Coordination of the Municipal Health Secretary of Sao Paulo (COVISA - SMS) - Health Surveillance Supervisors (SUVIS).

## DISCUSSION

Traffic accidents involving pedestrians represent a public health problem due to morbidity and mortality in Sao Paulo. With an expected population of 12.04 million of people in 2016, the coefficient of injury to pedestrians was 22.6 per 100 000 inhabitants. From 2723 records of traffic accidents with pedestrians, 62.7% involved males and 19.9% youth aged from 10 to 19 years. This observed data was similar to that found in other studies in which most victims were also male and young.^12^ ^-^ ^15^

In the age group from 0 to 19 years the rate of traffic accidents with pedestrians reached 36.9%, which indicates the vulnerability of children and adolescents as pedestrians due to their short stature and unawareness of traffic danger. This population does not have sufficient vision capacity above vehicles and also is not properly visualized by drivers at the rear of cars. In addition, children are still developing their psychomotor state, moving towards the achievement of a complete balance and dexterity, including factors of anatomy, physiology, environment and social relation.^13^

Given this data, traffic education, especially for children and youth is one of the instruments that can contribute to the reduction in medium and long-term accident rates. Effectively safe traffic can be achieved when citizens become more aware of their civil responsibilities and more respectful to the rights of others. Society will have citizens who develop these values ​​if, from an early age, children and adolescents are educated to become conscious pedestrians and drivers.^16^

Considering the elderly population (over 60 years), the data analyzed indicated a rate of 12.8% cases of traffic accidents with pedestrians, suggesting the need for a closer look at this population. Elderly are backwards to children and adolescents since they lose their sensory, balance, muscular and intellectual capacity, characterized by physiological and functional decline, thus becoming increasingly vulnerable.^13^ In the elderly between 60 and 74 years old involved in the accidents, approximately 25% evolved to death. For the population over 75 years, this rate may reach 50%.^4^

Traffic accidents with pedestrians are very violent. The greater physical vulnerability of the elderly population contributes to increased lethality. They represent an absolutely unequal shock, which can lead to serious injuries, even when vehicles are travelling at low speeds.^17^ In this way, it is necessary to stimulate the formation of attitudes of respect to elderly pedestrians with education and traffic awareness, as well as to invest in the construction of accessible and inclusive urban lanes that allow the right of universal mobility with safety. 

Analyzing the time of the day of recorded traffic accidents with pedestrians, there is a higher incidence (44.6%) of accidents in the afternoon, which suggests the relation between occurrences and a greater circulation of people. In this period, there is an increased flow of school children through the streets, due to the end of classes and of workers who have left their labour activities. According to Almeida et al.,^18^ accidents occurring at night present high gravity and can be related to less traffic on the streets, which allow the speeding of cars. In addition, the combination of alcoholic beverages and driving contribute to greater lethality of trampling.

The data analyzed suggest that there is no relationship between alcohol and drug use by pedestrians with the risk of trampling. Only 6% of registered cases were related to victims who consumed alcohol and 1.8% used some type of illicit drug.

According to Table 2, it is observed that data indicates the deficiency to record the location of the traffic accidents. With 85.6% of ignored locations, it is not possible to inquire whether there is a direct relationship between traffic accidents in regions that are more susceptible to being hit. In this way, public authorities will not have a baseline to focus on prevention of pedestrian accidents location of a higher risk.

The rate of traffic accidents with pedestrians caused by cars and motorcycles in Sao Paulo, in 2016, reached 70.3% of the occurrences. According to the Brazilian Institute of Geography and Statistics - IBGE, the fleet of vehicles in Sao Paulo is approximately 5.4 million cars and 895 thousand motorcycles.^19^ Certainly, a large share of this fleet is concentrated in urban areas, thus leading to increased risk of traffic accidents with pedestrians. In this way, the need for adequate traffic education is reassured, such as respecting traffic signals and crosswalks by drivers and pedestrians, improving road signs, streets pavement and lighting, controlling speed ​​and vehicles that run red lights.

Regarding types of injuries, it is observed that there is a predominance of contusion and multiple traumatisms. It is evident once again that trampling is always characterized by an unequal shock that causes greater damage to the victims because they do not use safety equipment at the moment of occurrence. Despite the fragility factors of pedestrians, 75.6% evolved to hospital discharge and only 0.7% to death. In any case, the activation of rescue teams, pre-hospital and hospital care, rehabilitation treatments, time away from labour activities, and consequences of trampling represent social and psychological impacts, in addition to high costs for public administration and victims.

In traffic accidents with pedestrian prevention actions, besides the signs and adjustments of roadways, the occurrences of these accidents are also associated with a lack of attention or distraction of both drivers and pedestrians. 

According to Lennon et al.^20^ the act of crossing or walking on the streets represent the smallest part of the time, but they are associated with a high risk of being hit. Crossing the street is a complex exercise that requires high demand for perception and cognitive ability of the pedestrian. External factors, such as the use of Smartphone when walking, generate distractions and may interfere with instant decision processes. These factors may compromise the assessment of imminent danger, interfere with the perception of distance and approach speed of vehicles causing the pedestrian to make mistaken decisions that exacerbate the risk of being hit.

The analysis of the factors that interfere in the occurrence of traffic accidents is a complex procedure because they are numerous and interrelated. There must be an enlargement of the attention on the traffic accident phenomenon from analysis of its characteristics of street lanes, pedestrians and vehicles involved. This aspect highlights the importance of intersectoral practices to confront the problem.^18^

## CONCLUSION

This study revealed the importance of keeping correct records of the occurrences data, so they can guide the government for intensive education of schoolchildren, adolescents, adults, elderly people and, mainly, drivers of vehicles for the development of a healthy society and respect for traffic regulations. In addition, they will be able to promote urban improvements in signalling and street’s pavement, and maintain pre-hospital and hospital care teams in strategic locations to minimize the time between the accident and the victim’s care.
